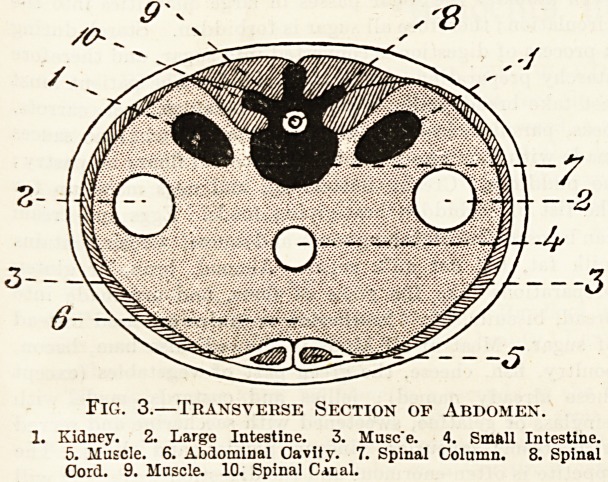# The Hospital. Nursing Section

**Published:** 1906-01-13

**Authors:** 


### The Hospital.

### Nursing Section.

Contributions for "THE HOSPITAL," should be addressed to the EDITOR, "THE HOSPITAL" NURSING SECTION, 28 & 29 Southampton Street, Strand, London, W.C.

No. 1,007.—VOL. XXXIX. SATURDAY, JANUARY 13, 1906.

### Notes on News from the Nursing World.

### THE NURSING QUESTION IN PARIS.

M. PAUL LUTARD, surgeon to the Paris Hospitals, contributes to our columns to-day an exhaustive account of the position of the nurses attached to these institutions. It will be observed that in his closing remarks M. Lutard says that the graduated nurses are not of the same social class as trained nurses in London, though they are fully equal to their work. This he attributes to the positive impossibility of inducing the daughters of professional men and merchants to entertain the idea of becoming nurses. He is hopeful, however, that by degrees their prejudice may be overcome, and there is no doubt that the authorities in the French capital are moving in the right direction. In fact, the substantial improvements which have been effected during the last ten years go far to justify this expectation.

### THE MATRONSHIP OF DURHAM COUNTY ASYLUM

WE understand that the Committee of the Durham County Asylum have appointed the assistant matron as successor to Miss F. M. Mitchell. The new matron began her career at the institution as one of the attendant nurses, and has been in the employ of the Committee for upwards of 20 years. The vacancy was advertised in several papers, and an age limit was mentioned. We conclude that the appointment made is in conformity with this limit.

### THE REPORTS OF POOR-LAW SUPERINTENDENT NURSES.

AT the last meeting of the Hampstead Guardians Miss Herford brought forward a motion to the effect that in future the superintendent of nursing should present her own report to the Guardians. In order to disarm hostility on the part of the Master of the Workhouse, she added to her motion a provision that the report, before presentation to the Guardians, should be submitted to him, and also that he should be at liberty to be present on the occasion when the report was considered. The motion was intended to take the place of two resolutions adopted by the Guardians in December 1901, which imposed upon the Master the responsibility of presenting the report book of the superintendent of nursing fortnightly to the Guardians, and recognised the book as "the only regular channel of communication" between the superintendent and the Guardians. Several of the Guardians strongly opposed Miss Herford's proposal, mainly on the ground that it would lead to a system of dual control, and in the end it was rejected. We do not see any particular objection to the superintendent nurse being allowed to present her own report to the Guardians.

### A MIDWIFE'S NEGLECT.

REFERENCE was made a few weeks ago in our columns to the fact that a midwife in Australia had been committed on a charge of manslaughter. This person, a married woman, has since been tried in the Supreme Court at Melbourne, the chief witness for the prosecution being the medical man who made an autopsy, and, having expressed the opinion that the death of the deceased had resulted from hæmorrhage after confinement, added that proper care and skill on the part of the midwife could have checked the hæmorrhage and prevented fatal consequences. For the defence it was contended that the midwife had done her best, and had vainly urged that a doctor should be called in. Ultimately, the jury, after deliberating for three hours, came to the conclusion that the accused showed neglect, but not gross neglect, and therefore they found a verdict of not guilty. The moral for midwives everywhere is obvious. If a case become serious the services of a medical man should be secured, whether the patient wishes it or not.

### THE QUESTION OF PAUPER ATTENDANTS.

THE urgent necessity of action at the North Dublin Union Workhouse was attested last week at the meeting of Guardians by Dr. Biggar, medical inspector under the Local Government Board, who, having dilated on the cruelty sometimes used by the pauper attendants, said that at present the nursing staff was not even large enough to supervise these persons. Dr. Kenny insisted that the nursing staff should be five times as large as it is, and after hearing his speech and that of Dr. Biggar, a majority of the Guardians decided that a committee with the help of the medical officers should draw up, as soon as possible, details of a nursing scheme with the view of effecting the changes required. We hope it will be recognised that no scheme can be thoroughly satisfactory which does not provide for the entire abolition of pauper attendants.

### THE MILITARY NURSING SERVICE.

WE are officially informed that Miss M. Russell, R.R.C., matron in Queen Alexandra's Imperial Military Nursing Service, has been transferred from Alton to the Military Hospital, York. Miss J. E. Dods, sister, from Alton; Miss L. E. Mackay, sister, from Alton; and Miss L. M. Toller, sister, from Portsmouth, have been transferred to Queen Alexandra's Military Hospital, Millbank. Miss K. A. Allsop, staff nurse, has been transferred from Aldershot to the Royal Herbert Hospital, Woolwich; Miss E. M. Bickerdike, staff nurse, from Alton to the Military Hospital, Portsmouth; Miss J. G. Dalton and Miss M. German, staff nurses, have been appointed to the Military Hospital, Gosport; and Miss G. S. Jacob and Miss F. M. Tosh, staff nurses, have been transferred from Alton to the Connaught Hospital, Aldershot. The appointments of Miss H. Hartigan, Miss H. J. Hepple, Miss E. M. Lang, Miss H. M. E. Macartney, Miss E. M. Rentzsch, Miss S. Richards, and Miss K. Roscoe as staff nurses have been confirmed.

### DEATH OF A CRIMEAN NURSE.

ANOTHER devoted woman who served under Miss Florence Nightingale in the Crimean War has passed away. Mother Mary de Chantal, formerly Miss Mary Louise Huddon, who died at the Convent, Wigton, last week in her 82nd year, had been a Sister of Mercy for 55 years. She entered the Convent of Mercy at Bermondsey in 1851, and was one of the religious sisters who, for 18 months, helped Miss Nightingale to tend the sick and wounded soldiers. Soon after her return she attached herself to the convent at Wigton, and there for 48 years she laboured, earning the esteem and affection of everybody with whom she came in contact. In 1897 Queen Victoria commanded Mother de Chantal, with two other sisters, to Windsor Castle, and bestowed upon her the decoration of the Royal Red Cross in recognition of her devoted services in the East. The honour came a very long time after the services rendered, but it was greatly appreciated by the recipient, who knew that it was not the late Queen's fault that it had not been bestowed before.

### FOR THE BENEFIT OF AUSTRALIAN NURSES.

WE understand that the garden fête held at the Government House, Adelaide, during October was a remarkable financial success. After payment of all expenses the South Australian branch of the Royal British Nurses' Association, the District Nurses' Association, and the Queen's Maternity Home, Adelaide, have each benefited to the extent of £1,000. This is an achievement on which the promoters at the Antipodes may fitly be congratulated.

### THE FIRE AT ADDENBROOKE'S HOSPITAL.

As soon as it was discovered on Saturday morning that a fire had broken out in the roof of the centre block of the Addenbrooke's Hospital, Cambridge, the orders were given to the patients in the wards near, namely, "Victoria," surgical women, "Humphrey," babies, and "Hatton," medical women, to be placed on stretchers preparatory to their removal to other wards. This was safely accomplished by the medical and nursing staff, assisted by voluntary helpers, the patients being carried down the outside staircases to the men's wards. Some were placed on empty beds, others on the floor, and the children two and three in one bed. The patients who were able to rise from their beds were all got up, as at one time there was danger of the fire spreading to the men's wards. But there was no panic of any kind; the patients' dinners were fetched from the kitchen by the nurses and others, the cook serving them out standing on a box, as the kitchens were quite 12 inches in water. The four-hourly temperatures were taken and medicines were given round as though a fire was an everyday occurrence. Nurses who could be spared from the wards helped with the hose, and in clearing away furniture, etc., from the proximity of the fire, standing and moving in pools and showers of water. Soon after 3 P.M. the order was given to take the patients back to the wards, and by 4 they were all having their tea as usual. Considerable damage was done to the centre block and the corridors, but excepting wet feet and damaged shoes, neither the nursing nor the domestic staff were any the worse for their experience; and while all of them worked splendidly, none got excited or hysterical.

### ENTERTAINMENT AT CHARING CROSS HOSPITAL.

A VERY pleasant entertainment was given at Charing Cross Hospital on the evening of January 3 by the presidents and sisters to the patients and nursing staff. In the out-patients' hall, prettily decorated for the occasion, a stage was erected, tastefully draped with green, and here a varied and excellent programme was gone through. The patients who were well enough to be present were seated in the front rows, and appeared to enjoy themselves immensely. It was evidently a popular function, as the large hall was crowded, and there were many standing at the back. During an interval in the middle of the performance, tea, cake, crackers, and chocolate were handed to the patients; while at the close of the evening tea and coffee were served to the guests in the dispensary waiting hall. Perhaps the most appreciated item on the programme was an exhibition of legerdemain by Mr. Charles Bertram, whose manipulation of the cards and the quite magical way in which he caused them to appear and reappear called forth rounds of applause. Miss Robinson sang two little songs by Liza Lehmann very sweetly, and the duet by Miss Wulff and Mr. Turner caused much amusement. Miss Helen Mott, besides playing two solos on the violoncello with great skill, contributed a humorous recitation by Kate Douglas Wiggin. The dramatic sketches by the Rev. Harold F. Davidson were listened to with keen attention. These were but a few among the many items of an attractive programme, which kept the audience amused from 7 until 10 o'clock.

### CHELSEA HOSPITAL FOR WOMEN.

THE festivities at the Chelsea Hospital for Women were brought to a close by the annual entertainment for the patients and nurses, held on Saturday afternoon. Among the various ways of celebrating Christmas may be mentioned the carol singing by Sunday-school children brought by Miss Roome on December 27, and the patients' tea on the 31st, when the ladies' orchestra supplied by Lady Moss Edwards and Miss Manning gave much pleasure to their hearers. The Ladies' Committee provided the funds necessary for the decoration of the wards and other expenses. On Saturday the Lazzaroni Trio of Neapolitan Singers gave an amusing performance to all the patients able to attend, and cleverly contrived to introduce great variety by alternating songs, dances, mandolin solos, trios, etc. Presents were afterwards distributed to all the patients.

### NURSES' ENTERTAINMENT AT THE BOLINGBROKE HOSPITAL.

ON Monday evening there took place at the Bolingbroke Hospital, Wandsworth Common, quite the most successful nurses' entertainment held in recent years. The out-patient department was prettily decorated and tastefully arranged. The guests, who crowded the hospital, showed themselves quite ready to enjoy to the utmost the very excellent programme prepared for them. In fact, the note of general good fellowship and enjoyment seemed to predominate to a striking degree. There was an excellent band in attendance, and Mr. Charles Bertram gave an admirable performance. A lady palmist, snugly ensconced in a little room, was also busily engaged during the evening. A demonstration of high-frequency apparatus was given by Mr. Cox, and there was a comprehensive display of instruments and appliances by Messrs. Maw, Son and Sons. Among the most interested of the company was Canon Erskine Clarke, but, contrary to expectation, Mr. John Burns was not able to be present.

### NURSES ON THE STAGE.

A feature of the season's festivities at the Newcastle-on-Tyne Workhouse Infirmary was the erection of a stage in one of the wards. The programme consisted of songs, carols, a couple of tableaux, and the representation of the comedy "Brown with an E." The parts in the play were taken by six of the nurses, and they acquitted themselves remarkably well.

### MATERNITY HOME AT WINDSOR.

In connection with the Princess Christian Nursing Institution at Windsor, it is proposed to establish a small maternity home. This addition will be the direct and necessary outcome of the work which has been carried on by the nurses of the institution among poor women in Windsor, Eton, and Clewer, and which had its origin in the desire of the Princess to assist the women whose husbands fought for their country in the South African War.

### RURAL MIDWIVES' ASSOCIATION.

Under the auspices of the Rural Midwives' Association a children's variety entertainment was given on the afternoon of Thursday last in aid of its funds. Queen's Gate Hall was crowded with friends and wellwishers, which now has 57 of its midwives at work in 15 different counties, and hopes to increase this number considerably after the approaching examination. The long programme provided afforded much pleasure and amusement. It included a comedietta, entitled "The First Anniversary," a magic lantern entertainment and a conjurer, recitations both humorous and otherwise, dances, folk-lore stories of birds and beasts, songs, and the performance of a mandoline and guitar band.

### GLASGOW ROYAL INFIRMARY.

The excellent custom of the Lord Provost of Glasgow and his wife meeting the nurses of the Royal Infirmary was observed, as usual, last week. In his speech on the occasion, the Lord Provost said that he did not think that he and his wife could begin the new year in any better way. He went on to contrast the position of affairs at the period their late esteemed medical officer, Dr. Russell, found it hard to improve the health of the citizens, owing to the difficulty of securing an efficient and well-trained band of nurses, with the present time, when medical men can always rely upon obtaining the assistance they need. The good wishes of the Lord Provost were reciprocated on behalf of the nurses and staff of the infirmary by the chairman, and subsequently Sir Samuel Chisholm briefly addressed the nurses.

### A PLUCKY CARLISLE NURSE.

It is not only in the discharge of their duties in attending upon the sick that nurses are exposed to the risk of personal violence. At the Carlisle City Quarter Sessions last week a young man was charged with assaulting a nurse while she was walking along the footpath near the river Eden. He first asked the time, and after she had told him, sprang at her. She struggled frantically with her assailant, broke her umbrella over his head, succeeding at last in driving him away. An attempt was made by counsel for the defence to prove that the man had no felonious intention, but the jury promptly returned a verdict of "guilty," and the Recorder, describing the case as "a gross and abominable crime," sent the accused to prison for two years—the utmost penalty he had the power to impose. It would have been more satisfactory if the ruffian could have been kept in penal servitude for five years.

### A MAGNIFICENT BEQUEST.

The late Mrs. Elder, of Glasgow, who two years ago gave away £200,000 to charities, has left the magnificent sum of £50,000 as an endowment fund for the Cottage Nurses' Training Home in connection with the Elder Cottage Hospital in Govan—which she founded—a commodious residence, and about half an acre of ground in South Avenue, Govan. Mrs. Elder took a deep interest in the progress of the movement for the multiplication of cottage nurses, and she was exceedingly anxious that they should obtain better training.

### SHORT ITEMS.

Out of the money received for admission to the State Apartments at Windsor Castle, the King has sent a donation of £30 to the Slough Nursing Fund, which provides trained nurses to visit the sick poor in their own homes.—Miss Wilhelmina Walker has been appointed a nursing sister in Queen Alexandra's Military Nursing Service for India.—On Tuesday Dr. and Mrs. Peill sailed from Southampton for Tsang Chow, North China, in order to undertake work under the London Missionary Society. Mrs. Peill, who was only married last month, was formerly Sister Louise Rhodes, having been trained at the North West London Hospital, and subsequently acted as night sister at the Mildmay Union Hospital, and matron at the Princess Christian Hospital in Sierra Leone.—Miss F. R. Herring has resigned her position as matron of Port Augusta special branch of study in Melbourne.—A dramatic concert is announced for Saturday evening next at 8 p.m. at the rooms of the Royal Society of British Artists, Suffolk Street, Pall Mall, by Mrs. Hale-Fitzgerald, assisted by Colonel Barrington Foote and other well-known musicians. She is the daughter of Mrs. Creighton Hale, author of "The Art of Massage," who herself was for some years a teacher.

### The Nursing Outlook.

"From magnanimity, all fear above;
From nobler recompense, above applause,
Which owes to man's short outlook all its charm."

### GRIEVANCES AND PUBLICITY.

THE question whether nurses are justified in making their grievances public comes intermittently to the front, and recent incidents invest it at the present moment with topical interest. There was the case of the nurses at Gartloch Asylum who complained, through the press, that their hours had been lately increased; and there was another of a more remarkable character, in which the Matron of Durham County Asylum addressed to this journal a communication making serious allegations of neglect against certain officials, letters supporting her complaint being subsequently forwarded to us from the entire staff of probationers. With regard to the latter we have waited for three weeks in the hope that the authorities of Durham County Asylum would endeavour to answer the charges preferred by the Matron. It is a curious fact that the local paper to which she addressed a communication similar to the one inserted in THE HOSPITAL, refused publicity on the ground that it is a rule of the editor not to interfere with public bodies and their officials. We consider it was a distinct advantage that the Gartloch nurses should have been able, by obtaining publicity for their grievance, to secure an explanation which has relieved the responsible officials from the odium of reducing the hours of leave, and has fastened the obligation upon the shoulders of the Lunacy Commissioners. In the case of Durham, where the matron and a medical officer who, according to their own showing—which must be accepted as accurate so long as it is not disputed—have been unjustly treated, as well as for that of the patients on whose behalf they took up the cudgels, and, in consequence, lost their appointments, we rejoice that a duty unrecognised by the local press was discharged by us.

Yet there are, it must be frankly admitted, many occasions when it is a mistake either to seek outside advice or to ask for outside sympathy in respect to grievances. Nurses have sometimes a tendency to accentuate the importance of trifles, and this tendency impels them to indulge in tittle-tattle which can never serve any good purpose, and often causes mischief to others without benefiting themselves. It cannot be expected that in an institution where a large staff is at work things will always go on smoothly. However pleasant a probationer may find people at first; however admirably matron and sisters may work together for a season, there will now and again be hospital jars. There is the other side of the shield, and a probationer who enters upon her period of training under the most depressing circumstances, with all sorts of grievances, real or imaginary, confronting her in the wards and in the home, invariably finds, if she will only exercise patience, that as the weeks and months go on the situation improves. Possibly the disposition to magnify grievances may prevail more widely among mental nurses, partly because some have not enjoyed the advantages of adequate training, and partly because of the wearing and more dangerous nature of the work. The steady improvement in their personnel, due to the elevation of the standard of mental nursing, will in time contribute to the preservation of equanimity in the presence of small discomforts. Nurses who feel that they have cause for dissatisfaction with their position should always, in the first instance, ask themselves how far they are to blame in the matter, and they should never be in a hurry to make accusations either against subordinates or superiors. If they are convinced that they have been victims of injustice, they should seek for redress at the hands of those whom they believe to be responsible for it before they take the world into confidence. The officials of an institution, from the least to the greatest, are bound by an unwritten code of honour, to refrain from taking any step which is calculated to bring a measure of discredit upon it unless, or until, they have exhausted their resources to get a wrong righted, or to gain a reform which is denied in the face of indisputable evidence of urgent need and obvious practicability.

But there may come a time when it is praiseworthy to seek publicity; when it would be blameworthy to refrain from pursuing the only means likely to remedy a crying injustice or prevent the commission of a deplorable blunder in policy. Hospitals are not private institutions, and our columns are always freely open to nurses in such circumstances as these. In fact, it is a pleasure to us to know that the fierce light of publicity thus afforded has frequently in the past had the curative effect desired. Petty quarrels, quibbles about minor details, passing errors of judgment, are more likely to be exacerbated than healed or rectified by proclaiming their existence. But rushing into print upon the slightest provocation is one thing, and falling back upon publicity in an emergency is quite another. There is no class which has forfeited this privilege; it is inalienable. It should be exercised sparingly, judiciously, with an adequate realisation of the issues involved, with courage and without ambiguity. Happy are those nurses who have no grievances! But for those who have, it is only fair that, in the last resort, the final court of appeal should be open, and the verdict of public opinion be procurable.

OK

### The Care and Nursing of the Insane.

By Percy J. Bailey, M.B., C.M.Edin., Medical Superintendent of Hanwell Asylum.

### I.—ANATOMY AND PHYSIOLOGY.

### 1. The Tissues.

The materials of which the body is built are called *tissues*. These differ widely from each other in their nature and properties to suit the different uses to which they are put. One—called bone—is dense and hard; another—muscle—is capable of changing its shape by contraction; another—nervous tissue—has the power of originating thought and of conducting impulses or messages from one part of the body to another; still another—gristle or cartilage—is tough and elastic. All these tissues are of more or less solid consistence, but the blood which plays so important a part in the body is a fluid tissue. The various tissues are ensheathed and bound together to form organs by what is called ordinary connective tissue, while the organs themselves are associated in groups or systems. Thus the nervous tissues of the body form the brain, spinal cord, and nerves, and constitute the nervous system. The heart, blood-vessels and blood, and the lymphatic vessels, comprise the circulatory system. The tube through which the food passes from the mouth to the rectum, and certain glands connected with it, the digestive system. The air-passages and lungs, the respiratory system. The skin, the cutaneous system. The kidneys and bladder and their ducts, the urinary system; and lastly the bones, cartilages, and ligaments, the skeletal system.

### The Cells.

All the tissues of the body are built up of minute structures called cells, " comparable to the bricks of a house." These in their simplest form consist of little sacs or bladders of living matter (called protoplasm) surrounded by a delicate outer covering and containing a differentiated particle within them called the nucleus. Such simple cells are found in the outer covering of the skin, but in most of the tissues they are greatly modified in form and size so as to enable them to carry on the particular work which is assigned to them. With regard to the functions which these cells perform, they may be compared to the individuals in a community, each of whom has his own particular work to do for the common good.

The science of anatomy treats of the structure and arrangement of these organs and systems of organs, while that of physiology describes the manner in which they perform their work or functions.

### Health.

So long as all the tissues and organs perform their functions so as to give the greatest possible amount of comfort to the individual, we say that a condition of health exists. This is the normal state, and the individual is not conscious of the existence of any of his organs. So soon as the functions of an organ are performed in such a manner as to give rise to discomfort or pain, in other words, when we become conscious of the existence of an organ—as when we suffer from toothache or palpitation of the heart—it indicates that the condition of health is departed from and that an abnormal state exists.

### Primary Divisions of the Body.

It is obvious that the human body consists of head, trunk and limbs. If the body were divided into a right and left half by cutting it through the centre (fig. 1), it would be seen that the tissues are

Fig. 1.—Longitudinal Section of the Body.

1. Mouth. 2. Pharynx. 3. Trachea. A. Thorax.—4. Œsophagus. 5. Bronchus. 6. Lung. 7. Heart. 8. Diaphragm. B. Abdomen.—9. Liver. 10. Stomach. 11. Duodenum. 12. Small Intestine. 13. Large Intestine. C. Pelvis. 14. Spinal Canal (for Spinal Cord).

so arranged that certain cavities the formed, these cavities being boxes which contain what we may call the vital organs. They are two in number, one of them—the anterior (or front) cavity—is contained entirely within that portion of the body which we call the trunk. Its dimensions are comparatively large, and it is divided by a transverse muscular partition, called the diaphragm, into an upper part—the thorax or chest—and a lower part—the abdomen or belly.

### THE THORAX.

The walls of the thorax are largely composed of bone (fig. 2), as we shall see, but those of the ab-

FIG. 2.—TRANSVERSE SECTION OF THORAX.

1. Spinal Cord. 2. Spinal Canal. 3. Spinal Column. 4. Gullet. 5. Lung. 6. Heart. 7. Rib. 9. Sternum. 10. Costal Cartilage.

dominal cavity (fig. 3) are almost entirely composed of muscles and fibrous bands. The lowest part, however, of the abdominal cavity is formed of a

FIG. 3.—TRANSVERSE SECTION OF ABDOMEN.

1. Kidney. 2. Large Intestine. 3. Musc'e. 4. Small Intestine. 5. Muscle. 6. Abdominal Cavity. 7. Spinal Column. 8. Spinal Cord. 9. Muscle. 10. Spinal Caial.

sort of basin whose walls are bony. This part of the abdomen is what is called the pelvis.

The chief contents of the thorax are the heart, lungs and gullet (œsophagus), together with many blood-vessels and nerves, and some lymphatic glands. These structures entirely fill the cavity.

### THE ABDOMEN.

The abdominal cavity contains the stomach and intestines and the liver and pancreas, the kidneys and bladder, of which the latter lies entirely within the pelvis, and a large blood-gland called the spleen. There are also within the abdominal cavity many blood-vessels, nerves and lymphatic glands. In women the pelvis also contains the uterus, or womb, and the ovaries.

The second, or posterior, cavity of the body is throughout enclosed within bone. The upper portion of this is the cavity of the skull, which is directly continuous with a narrow canal which lies within the backbone and which is called the spinal canal. This second cavity, therefore, lies partly in the head and partly in the trunk. Its function is to contain and to protect the brain and spinal cord (the central nervous system) which lie within it.

The general shape of the body is maintained by the bones and cartilages which form the groundwork or frame or scaffold upon which the softer parts are arranged. As a rule the bones are covered with the muscles and these again are surrounded by a layer of fat, while the skin is a protective covering for all. In every tissue of the body (except cartilage) there are abundant blood-vessels and nerves.

### 2. THE SKELETON.

The popular notion of a bone is something which is dried up and uninteresting—"as dry as a bone," as we say. This idea, as applied to a bone in its living state, is very inaccurate. Each bone is an organ made up of many parts. It is abundantly supplied with blood-vessels and nerves, and is covered with a tough fibrous vascular membrane called the periosteum, by means of which the bone grows in thickness. The outer part of the bone consists of dense tissue, almost like ivory, but the interior is filled up with bony material of a much looser, almost spongy texture. Many bones, especially the long bones of the limbs, are hollow, the cavity being occupied with a substance called marrow. Those parts of a bone which in the formation of movable joints come into contact with other bones, are covered with a layer of cartilage; here there is no periosteum.

### BONES

are composed of two quite different kinds of material (*a*) an earthy part, (*b*) an animal part. If any bone be soaked for a sufficiently long time in a dilute mineral acid, the earthy part is dissolved out and the animal portion (which we may call gelatine) only remains. The shape of the bone is retained, but it is now soft and pliable and can be bent in any direction. If, on the other hand, a bone be subjected to prolonged boiling, the animal portion is dissolved, only the earthy part being then left. Here again the shape of the bone is maintained, but it is now very friable and can quite easily be broken with the fingers and reduced to powder. A normal bone may be compared to a well-starched linen collar, the stiffness is due to the starch which impregnates the linen, when this is washed out the linen becomes limp, just as a bone does when the earthy salts are removed by the acid.

(*To be continued.*)

### To Nurses.

WE invite contributions from any of our readers, and shall be glad to pay for "Notes on News from the Nursing World," "Incidents in a Nurse's Life," or for articles describing nursing experiences at home or abroad dealing with any nursing question from an original point of view, according to length. The minimum payment is 5s. Contributions on topical subjects are specially welcome. Notices of appointments, letters, entertainments, presentations, and deaths are not paid for, but we are always glad to receive them. All rejected manuscripts are returned in due course, and all payments for manuscripts used are made as early as possible after the beginning of each quarter.

### The Nurses' Clinic.

### DIET IN DISEASE.

Diet is a very important factor in the treatment of all illness or disease. In the case of an acute attack the nurse will probably receive minute instruction as to diet—quantity and quality. But in many cases the medical man will allow the nurse to use her own discretion, and the only direction may be "Feed him up, nurse," or "Be careful with his diet." Then the nurse must rely on her knowledge as to the conditions requiring special diet and on her skill in preparing and serving suitable food. It is very difficult to lay down a hard and fast rule, as age, occupation, and special surroundings are often the cause why we are a little more or a little less strict in one case than another. In all disease we choose food in the first case which can be easily digested, always preferring those foods possessing the most nutritive value. In every case of disease digestion is impaired—in very acute illness it may even be suspended. Secondly, we give as much as possible in order to make up for tissue waste consequent upon the patient's state of health.

In any very serious illness nourishment must be conveyed in a liquid form, and the nurse, having been told to give a fluid diet, will have recourse first to milk—milk being the food of the greatest value. It contains all the elements required for nourishing and repairing waste tissue. But it may prove indigestible. If not digested curds form, and may be seen in the stools, or, if large curds are formed, they will be promptly vomited. Then it will be necessary to dilute the milk. It may be diluted with water—barley water or whey. A little lime water also may be added to each feed. If, however, the same symptoms occur—only minimised—then the milk must be peptonised. Liquor Pancreaticus and Fairchild's powders are generally the agents used, and full directions are given with each. A nurse ought to be very careful to stop the peptonising process before it has gone far enough to make the food bitter and unpalatable. Benger's food added to milk will often prevent curd formation. Plasmon renders the milk or beef-tea more sustaining. There are many preparations of Plasmon, such as Plasmon cocoa, Plasmon arrow-root, etc., all most palatable. Whey is made by boiling a pint of milk with two teaspoonfuls of lemon-juice and straining off the curds. It is easily digested, and made with white wine it is stimulating as well as digestible, and particularly useful in infantile diarrhœa. One wineglassful of sherry should be poured into half a pint of milk when the milk is boiling.

*Beef-tea* is useful because of its stimulating qualities and the salts in it, but it is not a food. It can be peptonised or made solid with isinglass. In the latter form, given cold, it is often a welcome variety, especially in cases of continued fever, where the rule is "little and often." Eggs contain a great deal of nourishment, and can be beaten up with milk, tea, coffee, or given raw with only brandy. The white of an egg well beaten and added to milk will make the milk taste more creamy, and is very nourishing, or, added to soda water and flavoured with lemon or brandy, forms a palatable nutritious beverage in acute illness.

*Alcohol* must be given with or without food, according to the medical directions. When it is given as a digestive it should be freely diluted and given with meals. If as a stimulant it must be given often, between feeds, and only diluted 1 part alcohol to 2 parts water. Tea, when allowed, is very refreshing, and made with milk is nourishing. As a flavouring for milk feeds tea, coffee, or cocoa may be used, as patients are very apt to complain that they are "so tired of milk." Water may always be given, and where there is much fever is continually asked for. It replaces the waste of water and flushes the system.

*Convalescent diet* can be greatly varied with a little thought, and yet be kept light and nourishing. Pounded fish or meat, with eggs and cream, tempting jellies, milk jelly, egg jelly, wine jelly custard, boiled or baked custard, junket with cream soufflés, etc., in small quantities. As the patient gets better an increase in the quantity of food is made, longer time is allowed between the meals, and chicken filleted, minced, boiled, or roasted may be given, alternating with fish and mutton. Until the patient is able to take exercise the meals should be carefully regulated in both quantity or quality, or digestion being upset a relapse may occur. The above scalê of diet may be used (with all necessary modifications) for any acute fever, gastric disturbance, tetanus, etc.

In certain diseases certain articles of food may be strictly forbidden, or, on the other hand, it may be necessary to try and force some kinds of food. Two very typical illnesses in which this is the case are diabetes and phthisis, and both allow of a liberal and varied diet.

*In diabetes* the sugar passes in large quantities into the circulation; therefore all sugar is forbidden. Starch during a process of digestion is converted into sugar, and therefore starchy preparations are to be avoided. The patient must not take bread, milk, sugar, fruit, potatoes, beet, carrots, peas, parsnips, broad beans, or Spanish onions; no sauces made with flour; no soups thickened with flour; no pastry; no puddings. Cream, butter, oil, and eggs make up for the list of forbidden heat-giving foods. Eggs and cream can be used for thickening soups and sauces. Cream contains milk fat, but not milk sugar. Almond, bran, or gluten preparations take the place of flour, and are made into bread, biscuits, etc. Saccharine or saxine are used instead of sugar. Meat of all kinds is allowed, also ham, bacon, poultry, fish, cheese, the green part of vegetables (except those already named); jellies and custards, made with isinglass or gelatine, sweetened with saccharine and served with cream; savouries, vinegar, pickles, and olives. The appetite is often enormous and the digestion weak. It will be the nurse's duty to regulate the food accordingly. Personal peculiarities must be studied. The conditions under which the digestion of food becomes inefficient or difficult are many and various, and each case must be dealt with accordingly. For the intolerable thirst of diabetes: Tea, coffee, cocoa (from nibs), with cream and saccharine; aerated waters; dry unsweetened wines and bitter ale may be given, or brandy or whisky.

*Phthisis* is due to the invasion of a bacillus, and therefore our great object is to improve the health, strengthen the tissues, and make the patient able to resist the attacks of the bacillus. Quantity, quality, frequency of meals, method of administration, must greatly depend upon the stage of the disease and the state of the digestion. In this case we must do our best to press certain foods. Fats and oils have been found to be very beneficial, so that fat in every form must be introduced into food and made digestible and palatable. Very often phthisical patients can digest more than the appetite allows of their taking, and every artifice of cookery must be brought into play, while nothing is omitted that will make the service of the food dainty and tempting. Some food before rising is essential. Warm milk with rum or brandy in it, or a cup of tea made with milk may be given. Breakfast to follow with fat bacon, dripping toast, buttered eggs, or a fried sole; tea with plenty of cream (coffee or cocoa if preferred), toast and butter. In the middle of the morning the patient should have an egg beaten up in milk, a cup of Benger's food, or junket with cream and sugar. A substantial midday meal to be as varied as possible. No twice-cooked meat, but fresh food cooked in various ways; milk puddings, suet pudding, etc. In the afternoon tea or cocoa made with milk, with thin bread and butter and a lightly boiled egg, sardine sandwiches, or a small piece of marrow toast. At 7 or 7.30 there should be a light nourishing meal of soup or fish, poultry or meat, and a sweet or savoury. Before going to bed warm milk, malted milk, gruel made with milk or Plasmon cocoa should be given. Wine, if ordered, or stout may be taken with meals, and brandy before rising or on going to bed.

In both these diseases the nurse will have much scope for variety and yet need to give a good deal of thought in "feeding up" the patients.

*Albuminuria* demands abstinence from meat, eggs, and stimulants, and in severe cases a milk diet is necessary. When the patient's condition improves, arrowroot and cornflour made with milk, milk-puddings without eggs, gruel, vegetable-soups, fish-soup and fish in various forms, gruel, jellies may be given; the latter must not have wine or eggs. Bread jelly as a convalescent food is nutritious and palatable, but it takes time to prepare. Stale bread—a thick slice—must be soaked five or six hours, the water strained off, and the remainder boiled gently for two hours and left to cool, when cold it will be in a jelly. If flavoured with a little lemon or mixed with milk and served with cream and sugar, it is much appreciated.

*Anæmic* patients require a generous diet, avoiding twice-cooked meats, pickles, vinegar, highly spiced food.

There is no more interesting or necessary study for nurses than diet in disease. Enough has been said to show how with all patients, in all illnesses, chronic or acute, much of the success of "treatment" is due to food being of the right quality or quantity. A patient should never be talked to about food. Observation will show a nurse what to avoid or disguise. If a patient refuses food it should be removed and presented later on in another form without comment. Dainty trays prettily arranged are a great assistance. Food should be prepared at a distance from the sick-room so that the patient cannot tell when the food is coming or what it is likely to be. A private nurse should always possess a good sick-room cookery-book and should be able to say how the food is to be prepared if she is not obliged to prepare it. The preparation of trays, etc., she will be wise to superintend personally.

### Incidents in a Nurse's Life.

### AN EPISODE IN THE MATERNITY WARD.

The probationer with a beaming important face tip-toed with exaggerated caution across the darkened ward, a precaution rendered null and void by reason of the deep-lunged roars emitted by the newly-born infant she carried in her arms. She paused at No. 8 cot, standing invitingly ready for its occupant, when the night nurse's voice made her turn.

"Not gone yet, nurse?"

"No, not yet, sister let me stay as Nurse Rowland is off with a sore throat. Look, nurse, isn't he a beauty? I expect you'll soon have a name for him."

The night nurse knew his name in a second; as she took the red-faced, kicking, screaming baby in her arms she recognised the original of a hundred painted counterfeits.

"Why, he's exactly like a baby on a Christmas card!" she cried; "you're my little Christmas card baby, do you hear? My wee, wee, Christmas card," gazing at the crumpled visage, and mechanically rocking to and fro as she spoke. "Now, nurse," with a return to more strictly professional tones, "don't you wait, I'll clear up, for if you don't make haste you'll be late for chapel."

To be late for chapel was indeed a venial sin in the eyes of good little Nurse Hill, so murmuring her thanks and hastily gathering together her possessions she hurried away.

An hour after perfect peace reigned in the little ward. Sister had gone to bed after giving the night report and bestowing one last look on her infant charges, and Mrs. S——, the mother of the "Christmas card" snored loudly beside her youngest progeny, whose cries had at last ceased, and with one fist in his mouth and the other thrown up above his head, slept the sleep of the just.

"Can't think how on earth you tell the little beggars apart," said a lank ward clerk sleepily, as he warmed himself in front of the roasting fire, while the night nurse quickly and noiselessly made up the "district midwifery bag" he was to take out to an urgent case.

"Nonsense," said nurse, fastening the bag and handing it to him, "they are no more alike than all plants or all dogs." She was country bred and the difference seemed very clear to her. "All alike, indeed," she murmured wrathfully to herself after the ward clerk had taken his departure, "as if anyone could possibly mistake 'Original Sin' for 'Her Majesty,' or darling little Mary." Poor "Original Sin" was certainly the least attractive of all the babies. He was the puny offspring of a boy and girl marriage, the sort one meets daily in the out-patient department, and who help to swell the annual death-rate of "infants under one year." His thin, skinny body, wizened face, and perpetual fretful wail were piteous in the extreme and no greater contrast could be found than his *vis-à-vis* "Her Majesty." She was certainly the biggest, fattest, sleepiest, and most placid baby ever born in Victoria Ward, and her voice was only uplifted just before meal times. "King John" came next, a motherless babe, but one who seemed to have found his particular world prepared to use him well, and little delicate Mary, the pet of the ward, whose nose was sadly put out of joint by the "Christmas card." Never was a baby more worshipped than this last arrival by everyone, from the great surgeon down to the cross little wardmaid, who would fervently cover the dimpled, tiny hand with kisses, a homage she never dreamt of rendering to "Her Majesty." The head day nurse invariably produced lovely robes for him out of her locked cupboard, and she would say daily to his mother in joke "When you go out, Mrs. S——, you must leave baby behind." Even sister, whose sense of justice forbade her to show favouritism, would make a point of seeing the "Christmas Card" kicking and crowing in his morning bath, yes, and dared to keep the aforesaid cross little wardmaid waiting for the dinner-list, while she watched the alluring performance. On the 10th day the mother got up, and the "Christmas Card" wore a pink ribbon in honour of the occasion. Two days later the night nurse reported that "Baby 8 did not sleep as usual, several times I found him with his eyes wide open," a strong tinge of anxiety underlying her even voice. The sister looked thoughtful and made a note, but worse was to come. The "Christmas Card" was suddenly seized with a convulsion, and towards evening a more violent one ensued, remedies seemed of little avail, the house physician looked grave and fetched his colleague and the two men consulted in low tones together, but nothing they tried seemed to be of any use. When the night nurse came on duty she found the baby had

**INCIDENTS IN A NURSE'S LIFE**—*continued.*

at last fallen into a heavy sleep. Alas ! this was the beginning of the end. The sixteenth day found "Baby Guy" (as he had been christened) just kept alive by unremitting tender care, still gripped at intervals by those terrible convulsions which left him so weak that one hardly expected him to survive from hour to hour. His mother had wept and gone home, but came every night from ten to eleven and sat beside the cot, not speaking or looking at him, but staring stupidly in front of her till the night nurse felt like crying out "Oh, love your baby, love him, you will have him for such a short time." Poor mother, who can say she did not feel love or grief? She had borne fourteen children and buried nine and hers was a hard life which had blunted all finer feelings. She had long ago forgotten how to caress or show affection, but her love dumbly expressed itself by her regular nightly attendance till the night nurse grew ashamed of her own hardness and her heart ached even more for the pathetic, mute figure than for the tiny, suffering mite who would so soon be taken to a Better Land.

The ward was very quiet now, "Original Sin" and "King John" had both gone out, so "Baby Guy" received almost undivided attention from his nurse; nevertheless on the 21st night, five minutes after she had tenderly replaced him in his cot, it was the mother who called in a husky voice, "Nurse, e's gorn, poor little dear, Gawd 'elp 'im!"

### The Threatened Strike at the Paris Hospitals.

By PAUL CH. LUTARD, Surgeon.

TOWARDS the end of the year some of the staff of the Paris Hospitals threatened to go out on strike. After an interview with M. Mesureur, Director-General of Public Assistance, things have quieted down, and everyone here hopes that the strike will not take place.

### How the Hospitals are Administered.

Before describing the exact position of the staffs of our hospitals, I should like to explain to the English public the way in which the Paris hospitals are administered. These hospitals are all governed by a department called "Public Assistance," which centralises all the funds received, these being: First, from its own special resources; second, subsidies from the Paris Municipal Council; third, private donations. This department, of very old standing, governs all the Paris hospitals, both general and special, and selects the personnel. Formerly, the Public Assistance made arrangements with the religious communities who undertook the care of the hospitals. These religious communities were superseded rather more than twenty years ago by a lay staff, except in two hospitals, Hotel-Dieu and St. Louis, where the sisters still remain in charge. The question, however, of replacing these by nurses is already under discussion. This great reform is the work of M. Bourneville, medical officer of Bicêtre Hospital and at one time a deputy, who has devoted his whole life to the reorganisation of the nursing system in Paris. Ever since 1882, the date of the secularisation of the hospitals, the department of Public Assistance has sought to improve the position of the nurses of the hospitals. Each year a certain sum of money has been expended to this end, but means being limited, it must be admitted that it has often been insufficient. When M. Mesureur was placed at the head of the Public Assistance Department he recognised that the position, moral, and material of the staff, both male and female, was far from being satisfactory. He consequently proposed making great changes, and on all sides he promised, both through the Press and in the hospitals, that he would give special attention to the subject of the nurses in the institutions.

### The Nursing Arrangements.

Starting from May 1, 1903, M. Mesureur succeeded in bringing about a complete reorganisation of the nursing arrangements of the hospitals. He established two distinct classes which had hitherto been merged into one, the one consisting of nurses, exclusively devoted to the care of the sick, both by day and night, known as the "personnel soignant," and the other of the domestic staff for general services, including those engaged in the various domestic offices, kitchen, linen-room, wash-house, as well as those employed in cleaning and looking after sanitary matters, the "personnel servant." By the same order the Director General unified the grades by substituting for the "surveillantes," "sous-surveillantes," and "suppléantes" the sole grade of "surveillante," comprising five classes, according to seniority or services rendered. He also reduced the working day to twelve hours, including meal times, instead of fourteen hours, and he increased the salaries of the whole staff.

### Regulation of Salaries.

From that date the annual salary has been regulated in the following manner: A probationer who wishes to enter the hospital is accepted as an auxiliary, and is called a "stagiare." She receives 400 francs (£16) a year, also board, lodging, laundry, and uniform. At the end of six months she obtains an increase of 50 francs (£2), and six months later, according to her capabilities, the department places her either among the class of "personnel soignant" if she has had instruction or possesses a diploma from some nursing school, or, if not, among the class of "personnel servant." In the first instance the nurse—almost always a woman—may be compared to a "trained nurse." She becomes successively third-class nurse (500 francs—£20), second-class nurse (600 francs—£24), and first-class nurse (700 francs—£28). After that stage she passes from the non-graduated staff of the hospital into the graduated ranks. These nurses, known as "surveillantes," are divided into five classes, in which the salary in each class is 100 francs (£4) more than in the preceding class, so that the nurses receive from 800 francs to 1,200 francs (£32 to £48) per annum. This latter figure is the maximum salary which a "surveillante" can obtain. In addition, if a "surveillante," by reason of her being married or for lack of accommodation in the hospital, etc., receives neither board, lodging, laundry, nor uniform, she is given over and above her salary an allowance of 1,300 francs (£52), which includes 350 francs (£14) for lodging, 750 francs (£30) for food, 200 francs (£8) for the other expenses. If a non-graduated nurse resides outside the hospital the allowances, in addition to the salary, are 1,000 francs (£40), which includes 240 francs (£9 12s.) for lodging, 650 francs (£26) for food, 110 francs (£4 8s.) for the other expenses.

### The Pension System.

Finally, M. Mesureur, wishing to provide for the old age of all the staff by means of a deduction of 5 per cent. of their salaries, insures their receiving a pension. These pensions, into the details of which I will not enter for fear of overburdening this article with statistics, vary according to the position of the recipient and the years of service. At the end of twenty years the pensions may be from 1,000 francs (£40) for a "surveillante" to 680 francs (£27) for a third-class nurse. I should add that if these salaries seem small, they nevertheless show a great advance over those received before 1903; they must, moreover, be considered satisfactory compared with the salaries obtained generally for women's work in Paris.

### Why the Strike was Threatened.

I now come to the question of the strike. It is closely connected with the salaries of which I have just spoken. The finances of the Public Assistance Department would not allow of these reforms being carried out for the whole of the hospital staff at the same time. The senior members of the staff were first dealt with, and consequently the "surveillantes" were favoured at the expense of those belonging to the non-graduated staff. It is these and, above all, the men belonging to the "personnel servant" class, who, having become discontented, have threatened menacingly to strike to obtain at once improvements which have been promised to them, but which they can only receive by degrees. Above all, they insisted on being neither boarded nor lodged in the hospital, but wished to receive the allowance of 1,000 francs (£40), details of which I have already given. They complained of being badly lodged—which is, unfortunately, generally true—and badly fed. This last complaint is, according to M. Mesureur, ill founded, and the Director-General asserts that his department purchases no articles that are not of the best quality. This would seem to be the case, since the food for the staff is the same as that for the patients, and the latter, as a rule, do not complain.

### M. Mesureur and the Malcontents.

The malcontents held a public meeting, after which they drew up a notice which was posted on the walls of Paris. Following these manifestations M. Mesureur had an interview with the representatives of the nursing staff. He had no difficulty in proving to them that he had already done much for the nursing staff of the hospitals, that he would continue to improve the lot of the domestic staff, but that he could only proceed gradually. He exhorted them to practise patience and moderation, and the delegates, in the name of their fellows, agreed to await the arrival of the promised reforms. Such is the state of affairs. In my opinion the strike will not take place. The malcontents are not the most numerous party. The majority are men who belong almost entirely to what for want of a better name I term the domestic staff ("personnel servant"). It does not seem likely that they will easily gain over the nursing staff ("personnel soignant"), which is almost entirely composed of women. The "surveillantes" and the "infirmière-panseuses" (trained nurses) are sufficiently intelligent to understand that it is to their interests not to forsake the patients. Besides, the timidity of their sex is an obstacle to their taking any such violent action as a strike.

### The Position To-day.

I think I have briefly sketched the exact position of the hospital staff in Paris—a position which is improving daily, materially and also morally, for as a whole the nurses are coming to be appreciated by the people. Unhappily, this has not always been the case in Paris, and it is partly the misfortune of the nurses themselves, who, from a social point of view, cannot be compared to English nurses. From a professional point of view our nurses who follow the courses of the five nursing schools are fully equal to their work; unfortunately, they are not of the same social class as those whom we admire in London. This fact is deeply regretted by physicians and surgeons alike. It has so far been positively impossible to induce the daughters of doctors, merchants, professors, etc., to entertain the idea of becoming nurses, so that these positions are chiefly filled by the daughters of working men and peasants. I trust that this state of things may in time cease to exist, and that, little by little, we shall see girls of gentle birth, who need or desire to earn their own living, embrace the nursing profession, and that thus, by degrees, the nurses of the Paris hospitals will resemble more closely those of similar charitable institutions in London.

### The Nursing of a Fractured Leg.

### EXAMINATION QUESTIONS FOR NURSES.

The question was as follows: Imagine yourself called upon to nurse a fractured leg (tibia and fibula) in a poor outlying cottage where the bed was flock, on a worn and loosely-stretched sacking. What arrangements should you make to keep the leg in good position? Remember that appliances and money are absent—and shops even—many miles distant!

### First Prize.

First of all I should look about to see what I could find in the way of a board to place under mattress. In most cottages, however poor, one could find either one or two shelves, which could be easily detached from wall and readjusted after use; or, failing them, a couple of small shutters, which could be easily removed from hinges. These I should place to come as near as possible from small of back to ankle and place firm pillow well under shoulders and back. If I had no one to help place boards in position I should proceed in this way (of course, I am concluding the doctor has put leg in splint).

First, I should get a stout sheet, failing that a coverlet, blanket, or even table-cloth, and fold lengthways to form draw-sheet. With that I should get patient as near side of bed on injured side as possible, then from other side should place boards as far under mattress as they would go; lift patient carefully on board side, and then steadying leg with one hand, very gently push boards a little further until they were well in position. Of course, it is much better to have two people, one to lift and the other to place boards, but I have found above way easiest both for patient and nurse when alone, and the leg in a splint.

If procurable I should put blanket folded double under draw-sheet to reach from middle of back to ankle, and draw both very tightly, securing firmly on either side with strong safety pins, which I always carry with me. I should place box with ends off over leg to keep clothes from pressing on it.

"Few."

### Second Prize.

If I were called to nurse a fractured leg (tibia and fibula) under the circumstances mentioned I should try to tighten the sacking, and if this were impossible should try to get boards or a door; or, failing these, a piece of wire netting and stretch across the bedstead. I should then arrange the flock as smoothly as possible and tie it to the netting (through and through) to prevent it getting into lumps.

Should fill two stockings with sand or tightly-packed straw and use as sandbags if these were not available.

"Glasgow."

### The Prize Winners.

"Few" gains the first prize, for she has grasped the necessity of making the best of but poor appliances; she also has good ideas with regard to moving the patient without assistance, but I should counsel the seeking the help of someone else rather than relying solely on one's own thews and sinews, someone is quite sure to be available; neighbours are kind to each other, and presumably there would be a wife, mother, or son or daughter in the house. "Glasgow" is successful with the second prize; her idea of the wire netting on which the old bed could be fixed is not bad, but I think experience would prove that it would very soon "sag" in the middle. The boards are the more dependable makeshift. The stockings filled with sand would prove very useful, or earth (previously baked) straw would not answer well because it is light. I should like to remind all nurses engaged in country district work of the merits of a large tea-tray to make a firm resting place for the injured leg. Turned upside down it is most useful, and one is generally found in the majority of cottages; not, indeed, for ignoble use, but as an ornament and background to the family Bible and group of woollen flowers and fruit.

HONOURABLE MENTION.

This is gained by "Sidla," "Sago," "Dot," and "Eileen."

QUESTION FOR JANUARY.

If called upon in district nursing or otherwise to arrange the diet of a person suffering from an exhausting disease, such as consumption, and if that person belonged to a labourer's family (consisting, perhaps, of six or seven people), whose wages did not exceed 18s., or thereabouts, per week, what should you advise as the cheapest and most nourishing scale of diet?

A caution as to the answers. This question was put more than two years ago, and the papers sent in disclosed the fact that nurses generally understand very little how small a sum is 18s. on which to bring up a family per week. Try to grasp that milk and eggs are expensive luxuries, even in the country, and as for cream, as advised by some competitors, that is a luxury—under the circumstances—not to be thought of for a moment. THE EXAMINER.

RULES.

The competition is open to all. Answers must not exceed 500 words, and must be written on one side of the paper only, without divisions, head lines, or marginal notes. The pseudonym, as well as the proper name and address, must be written on the *same* paper, and *not on a separate sheet.* Papers may be sent in for 15 days only from the day of the publication of the question. All illustrations strictly prohibited. Failure to comply with these rules will disqualify the candidate for competition. Prizes will be awarded for the best two answers. Papers to be sent to "The Editor," with "Examination" written on the left-hand corner of the envelope.

In addition to two prizes honourable mention cards will be awarded to those who have sent in exceptionally good papers.

N.B.—The decision of the Examiner is final, and no correspondence on the subject can be entertained.

Any competitor having gained three prizes within the current year shall be disqualified from taking another until 12 months shall have expired since the first prize was gained.

### Presentations.

DUMFRIES AND GALLOWAY ROYAL INFIRMARY, DUMFRIES.—Miss Julia Armstrong was presented by the staff and medical chiefs of Dumfries Infirmary with a gold bracelet and silver toilet requisites on the occasion of her resignation of her charge as sister of the medical and sanatorium wards. The presentation was made in the presence of the staff by the matron.

GLOUCESTER DISTRICT NURSING SOCIETY.—Miss Dudley, late Superintendent of the Gloucester District Nursing Society, has been the recipient of a gratifying testimonial from her Committee and friends in Gloucester. It consisted of a piano of walnut wood. From the present nursing staff and a few of her old nurses she received a gold curb bracelet, gold cross, eye-glass chain, shoe-horn, and a framed picture of "The Nativity."

SUNDERLAND UNION WORKHOUSE HOSPITAL.—On Christmas morning the nursing staff of the Sunderland Union Hospital once again had the pleasure of presenting to their Superintendent Nurse, Miss A. S. Pruett, a small token of their great regard, which took the form of a chased silver cake-basket.

### Practical Hints.

We welcome notes on practical points from nurses.

### RAISING THE FOOT OF THE BEDSTEAD.

IT has often been said that "life is made up of trifles." Certainly trifles have an important place in all lives, especially in those of the sick or weakly.

Elevating the foot of a bedstead may seem a very trivial thing, but I should like to mention a few of the circumstances in which I have found it beneficial—I was going to say "seen it work wonders"—for in more than one individual case it has really seemed to do this.

For patients suffering from fractures, etc., of the lower limbs, where extension is employed, it is, of course, routine treatment; but it will also be found of service in nearly all cases where it is customary to place the foot or leg on a pillow, such as wounds of the lower extremities or œdema of the same. The pillow always seems to sink down until it is useless, and—unless tied into place—to get where it is of little or no use. The pillow may still be used if found comfortable, but one feels that if the foot of the bed is raised it is not so necessary to be always shaking it up, and placing it in position.

Also with a helpless patient has a tendency to slip down in bed.

Where a water-bed is in use.

When giving a nutrient enema it will be more likely to be retained if the bed is raised before doing so.

In obstinate cases of constipation and in most cases of intestinal obstruction a purgative enema is more likely to prove efficacious if the foot of the bed is well raised before and lowered again some ten to fifteen minutes after administering.

Some cases of irreducible hernia become reducible if the foot of the bed is appreciably raised.

In cases of faintness and sudden collapse great benefit may follow. It has also a good effect in many cases of insomnia, especially if anæmia is present.

If there be a doctor in attendance I always secure his approval before causing the bed to be tilted, but there are very many hard-working mothers who are not invalids, but whose feet and legs swell from continuous standing and who suffer with varicose veins, varicose ulcers, etc. Anæmic girls often find their legs are terribly swollen; to say nothing of tired nurses themselves, including the newest probationer who is just discovering that she possesses feet. Each and all of these would derive very great benefit from having the bed they sleep in permanently raised nine or ten inches from the ground, at the foot.

For raising the bed, I use in an emergency, such as sudden faintness, choking from hæmorrhage, hernia, etc, two wooden chairs, one under each leg at the foot of the bed.

For permanent use, there are several forms of blocks to be procured at the various instrument makers. In the district I use parcels of bricks, one, two, or three, according to the height I require. These I tie together firmly with string and then wrap up as neatly as I can in brown paper, sealing the ends with wax. Many bricks are slightly hollowed in the centre on one side. I arrange that one of these shall come so that the foot of the bed may rest in the groove. Sometimes I get a joiner to make me a couple of blocks nine or ten inches high, with a base seven inches square, having a cup-like depression in the centre of the top for the castor to rest in.

### Everybody's Opinion.

[Correspondence on all subjects is invited, but we cannot in any way be responsible for the opinions expressed by our correspondents. No communication can be entertained if the name and address of the correspondent are not given as a guarantee of good faith, but not necessarily for publication. All correspondents should write on one side of the paper only.]

### THE ROYAL NATIONAL PENSION FUND.

"POLICY HOLDER 10152" writes: Will you kindly allow me to tender my sincere gratitude and thanks to Mr. Dick, Secretary of the Pension Fund, and also to the Secretary and Committee of the Junius Morgan Benevolent Fund, for the great kindness and help which they have meted out to me during the past few months. One only requires to become a policy holder and have practical experience of the Royal National Pension Fund to know its great value to nurses and to realise how much the authorities have their welfare at heart. I sincerely hope that there will be a great increase in the number of policies in 1906.

### OVER-WORK IN POOR-LAW INFIRMARIES.

"M. F." writes: There are continual complaints of the difficulty of getting nurses to accept Poor-law appointments, and of frequent resignations of the nurses. I do not think it is to be wondered at when one considers the conditions which some of the nurses work under. I know of a big London infirmary where the night nurses are responsible for 90 to 100 patients, many of these being really acute cases with delirious and occasionally lunatic patients. The night nurses are on night duty for three months, and during that time they do not have a night off. I think most nurses know the strain of prolonged night duty, even with an occasional night in bed, not to sympathise deeply with those who have this length of time on night duty without ever being free for a night. Besides, what can one nurse do for 90 patients, and these not only in separate wards, but on different floors, with a long flight of stairs between? The continual strain of trying to be in two places at once must be very bad indeed, and also the impossibility of doing one's duty to the sick under one's care. If it is necessary in some infirmaries to have one nurse to 28 patients, what can happen to the nurse who has 90 sick human beings under her care? Is there any wonder that she gives up in despair and resigns in favour of some other appointment where it is more easy to carry out the traditions of nursing?

### Appointments.

[No charge is made for announcements under this head, and we are always glad to receive and publish appointments. The information, to insure accuracy, should be sent from the nurses themselves, and we cannot undertake to correct official announcements which may happen to be inaccurate. It is essential that in all cases the school of training should be given.]

CHELTENHAM GENERAL HOSPITAL.—Miss Florence Alexander Harris has been appointed Sister. She was trained at the Newport and Monmouthshire Hospital, and has since been senior staff nurse at Bromley Cottage Hospital, Kent, and charge nurse at the Royal Victoria Hospital, Bournemouth.

CHRISTCHURCH HOSPITAL, NEW ZEALAND.—Miss Alleyne has been appointed charge nurse. She was trained at the Adelaide Hospital.

GREAT YARMOUTH HOSPITAL.—Miss M. E. Warhurst has been appointed sister. She was trained at Rotherham Hospital and Dispensary, and has since been staff nurse at the Royal Hospital for Diseases of the Chest, City Road, London.

HOSPITAL FOR INCURABLE CHILDREN, SWISS COTTAGE.—Mrs. Tipper and Miss Fearn have been appointed staff nurses. Mrs. Tipper was trained at Glasgow Royal Infirmary and has since been charge nurse at the Provincial Hospital, Port Elizabeth, Cape Colony, and house-mother at the Ophthalmic Schools, Swanley. Miss Fearn was trained at Southwark Infirmary, East Dulwich.

INFECTIOUS DISEASES HOSPITAL, BOOTLE.—Miss M. A. Gordon has been appointed sister. She was trained at Edinburgh Royal Infirmary.

LEWES NURSING ASSOCIATION.—Miss Alice Wood has been appointed lady superintendent of the Nurses' Home. She was trained at the Sussex County Hospital, Brighton, and has since been engaged in private nursing, and for some years she has acted as matron's *locum tenens* at the Miller Hospital, Greenwich, and at Victoria Hospital, Lewes.

LLANELLY HOSPITAL.—Miss C. A. Edwards has been appointed staff nurse. She was trained at the Steyning Union Infirmary, Sussex, and she has since done private nursing. She holds a certificate in midwifery.

NATIONAL HOSPITAL, QUEEN SQUARE, BLOOMSBURY.—Miss M. R. Keith has been appointed home sister. She was trained at the Royal Alexandra Infirmary, Paisley, and has since been private nurse in Glasgow, charge nurse at the Cunningham Combination Hospital, Ayrshire, head night nurse at the Royal Alexandra Infirmary, Paisley, sister at the County Hospital, York, and night sister at the National Hospital, Bloomsbury.

NEWCASTLE-UPON-TYNE UNION INFIRMARY.—Miss Gertrude R. Taylor has been appointed charge nurse. She was trained at Crumpsall Infirmary, Manchester and was subsequently night sister at Mill Lane Hospital, Liscard. She has since done private nursing in connection with the West Riding Nurses' Co-operation, Rotherham. She holds the certificate of the British Gynæcological Society.

QUEEN'S JUBILEE HOSPITAL, EARL'S COURT.—Miss Lilian Fulton has been appointed Matron. She was trained at St. Bartholomew's Hospital, London, where she was afterwards assistant housekeeper. She has since been Matron of Noble's Hospital, Isle of Man.

RICHMOND WORKHOUSE INFIRMARY.—Miss Amy Maskell has been appointed charge nurse. She was trained at Poplar and Stepney Sick Asylum, and has since been assistant nurse at the Cuckoo Schools, Hanwell, and at Stepney Union Infirmary.

ST. LUKE'S HOSPITAL, HALIFAX.—Miss Eva Jones has been appointed assistant matron, and Miss Ethel Mary Williams night superintendent. Miss Jones was trained at Mill Road Infirmary, Liverpool, and in midwifery at the Liverpool Lying-in Hospital, where she afterwards became sister. She has since been sister at the Parkfield Nursing Home, Liverpool, taken matron's holiday duty at the Samaritan Hospital, Liverpool, and been night superintendent at St. Luke's Hospital, Halifax. She holds the certificate of the Central Midwives Board. Miss Williams was trained at Camberwell Infirmary, where she has since been staff nurse, sister of the male surgical and phthisical blocks, and theatre sister.

SOUTHALL-NORWOOD SANATORIUM.—Miss Cook has been appointed nurse matron. She was trained at the Chorlton Infirmary, and has since been charge nurse at one of the hospitals of the Metropolitan Asylums Board, night superintendent at Glasgow Fever Hospital, and sister at Coventry Fever Hospital.

STOCKTON AND THORNABY HOSPITAL.—Miss L. N. Sheppard has been appointed sister in charge. She was trained at the Royal South Hants and Southampton Hospital.

STROUD HOSPITAL.—Miss Margaret Josiah has been appointed charge nurse. She was trained at the Norfolk and Norwich Hospital, Norwich.

### Notes and Queries.

**REGULATIONS.**

**The Editor is always willing to answer in this column, without any fee, all reasonable questions, as soon as possible.**

**But the following rules must be carefully observed.**

**1. Every communication must be accompanied by the name and address of the writer.**

**2. The question must always bear upon nursing, directly or indirectly.**

**If an answer is required by letter a fee of half-a-crown must be enclosed with the note containing the inquiry.**

*Training.*

(105) I am a shop assistant, but should like to go in for hospital nursing, but am not very well educated. Do you think it possible that I might succeed with hard work, as I am very strong, or is it always necessary to have a good education to become a nurse?—*A. B. C.*

"A good education" is nearly always necessary; but you might write to the matrons of some of the Poor-law infirmaries and ask for application forms, at the same time inquiring when there will be a vacancy. Consult "How to become a Nurse," published by the Scientific Press, Ltd., for full information.

*Liverpool.*

(106) Can you tell me if there is a hospital or infirmary in or near Liverpool where I could go as a probationer?—*G. T.*

Write to the matron of the Royal Liverpool Infirmary, or the matron of the Royal Southern Hospital, Liverpool.

*Australia or New Zealand.*

(107) I am anxious to do private nursing abroad in either New Zealand, Sydney, or Melbourne. Can you give me information with regard to institutions where certificated nurses are employed?—*Higgs.*

Write to the lady superintendent of the Melbourne Trained Nurses' Home, 384-386 Albert Street East, Melbourne. Only certificated nurses are employed by this association.

*The Question of a Fee.*

(108) I am a midwife, and one of my patients engaged me to attend her twice a day the first week and one visit a day the second week. She also engaged a doctor. A few days ago she called to ask me if I could tell her of a person who would undertake to do the housework, as her mother—who was to have done so—was unable to come. I sent her a very capable person, but the following day I received a letter from the patient saying that she would have to cancel her engagement with me, as she had come to the conclusion she could not afford to pay doctor, nurse, and person for housework. I should be pleased if you could tell me what portion of the fee I can claim.—*Nurse.*

As the patient broke the engagement, you are legally entitled to the whole fee. Perhaps, however, if the patient is poor, you can arrange to accept half.

*Asylums.*

(109) Are there any asylums, private or otherwise, in British Columbia or Canada? Will my not having the medico-psychological certificate deter me from getting an engagement out of England? I have had three years and a half asylum and one year and a half private experience, but hold only the St. John's Ambulance certificate.—*W. C.*

Several, but you would find that not possessing the Medico-psychological certificate would be a handicap to you in trying to obtain mental cases, and there are plenty of home-trained mental nurses in both the colonies you mention.

*Home.*

(110) Do you know of any home where aged and enfeebled gentlewomen are received on payment of a small annual sum by relatives? As a young, unmarried man in lodgings, I do not feel properly qualified to look after my grandmother satisfactorily, but could only afford to pay about £25 per annum.—*H. B.*

Write to the Hon. Secretary, the Frithville Homes for Reduced Gentlewomen, 57 The Grove, Hammersmith, W., or advertise in The Hospital. A retired nurse might be glad to look after your relative.

**Handbooks for Nurses.**

| | Post Free. |
|---|---|
| "A Handbook for Nurses." (Dr. J. K. Watson.) ... | 5s. 4d. |
| "Nurses' Pronouncing Dictionary of Medical Terms" | 2s. 0d. |
| "Art of Massage." (Creighton Hale.) ... ... ... | 6s. 0d. |
| "Surgical Bandaging and Dressings." (Johnson Smith.) ... ... ... ... ... ... | 2s. 0d. |
| "Hints on Tropical Fevers." (Sister Pollard.) ... | 1s. 8d. |

Of all booksellers or of The Scientific Press, Limited, 28 & 29 Southampton Street, Strand, London, W.C.

### For Reading to the Sick.

THE POWER OF PRAYER.

We kneel how weak, we rise how full of power!
Why therefore should we do ourselves this wrong,
Or others—that we are not always strong,
That we are ever overborne with care,
That we should ever weak or heartless be,
Anxious or troubled, when with us is prayer.
And joy and strength and courage are with Thee?

*Trench.*

To the lowliest and feeblest of God's children is given the privilege of prevailing prayer. We may lay hold upon God's strength. We may make intercession for others and call down upon them the most gracious blessing. We may unlock storehouses of divine goodness and gather treasures at will. All things in earth and heaven are within the reach of him who prays.

Much instruction is found in the order of the petitions of the Lord's Prayer. We are apt to think first of our own frets and worries, our own wants and desires, when we come to God and to begin at once to pour these into His ear. But it is not thus that we are taught by our Master to do. Half of the Lord's Prayer is finished before there is a word about the earthly needs of him who is praying.

It is very comforting, however, as we go on, to find that there is a place in the Master's model of prayer for the commonest wants of daily life; that we may ask our Father even for the bread which our body needs. Only we should never forget to keep self and all personal wants and troubles in their true place, far secondary to our longing and asking for the things of God. Only that prayer is effectual in the largest measure which puts the honour of God and the interests of God and His cause above all else in its desire.

All the petitions require us to unite others with ourselves. We must come to God as "Our Father," we must enter at the right gate, the children's gate. We must approach God saying, "Our Father." This means that we must come to God in prayer as His children and not doubt that as quickly as the words "Abba, Father," are spoken, the door will open to us.

*Dr. J. S. Miller.*

We pray for childlike hearts,
For gentle, holy love;
For strength to do Thy will below,
As angels do above.

Let me find in Thy employ,
Peace that dearer is than joy;
Out of self to love be led
And to heaven acclimated
Until all things sweet and good,
Seem my natural habitude.

*J. G. Whittier.*

## Figures and Tables

**Fig. 1. f1:**
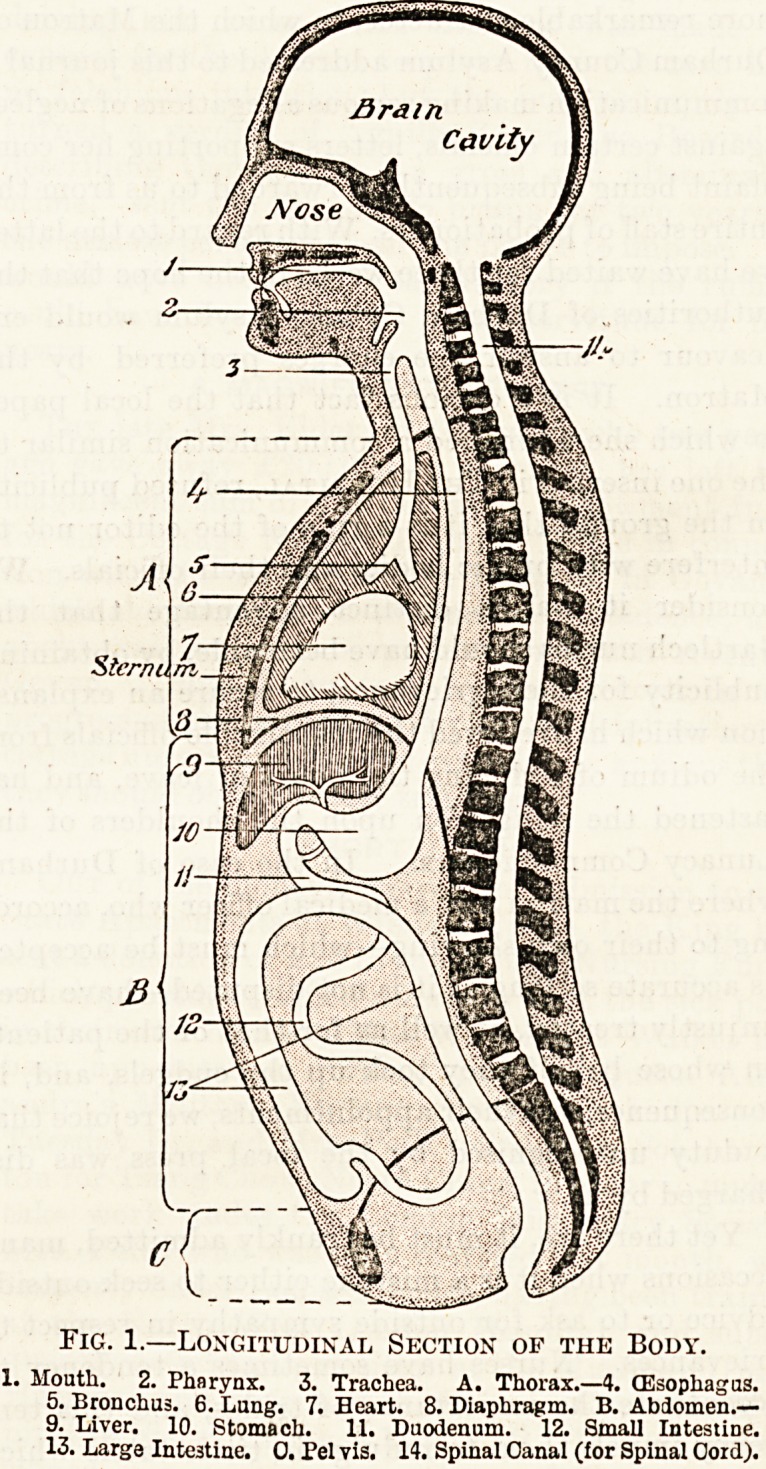


**Fig. 2. f2:**
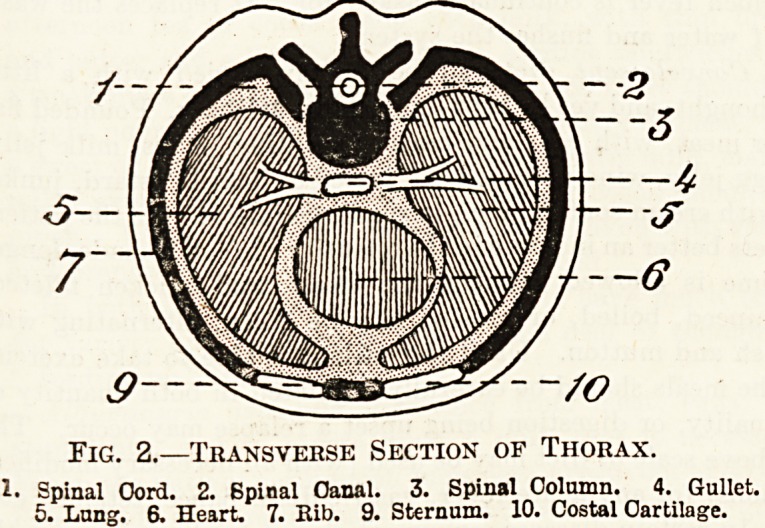


**Fig. 3. f3:**